# Effects of oral administration of *Bifidobacterium animalis* subsp. *lactis* HN019 on the treatment of plaque-induced generalized gingivitis

**DOI:** 10.1007/s00784-022-04744-y

**Published:** 2022-10-28

**Authors:** Yara Loyanne de Almeida Silva Levi, Marcella Costa Ribeiro, Pedro Henrique Félix Silva, Giselle Aparecida Silva, Sergio Luiz de Souza Salvador, Sérgio Luís Scombatti de Souza, Renato Casarin, Arthur Belem Novaes Júnior, Mario Taba Júnior, Daniela Bazan Palioto, Heitor Marques Honório, Michel Reis Messora, Flávia Aparecida Chaves Furlaneto

**Affiliations:** 1 Department of Oral Surgery and Periodontology, School of Dentistry of Ribeirao Preto, University of Sao Paulo – USP, Ribeirão Preto, São Paulo, 14020-150 Brazil; 2 Department of Clinical Analyses, School of Pharmaceutical Sciences of Ribeirao Preto, University of Sao Paulo – USP, Ribeirao Preto, , São Paulo, Brazil; 3 Department of Prosthodontics and Periodontics, School of Dentistry, Campinas State University – UNICAMP, Piracicaba, São Paulo, Brazil; 4 Department of Pediatric Dentistry, Orthodontics and Public Health, School of Dentistry of Bauru, University of São Paulo - USP, Bauru, , São Paulo, Brazil

**Keywords:** *Bifidobacterium lactis*, Gingivitis, Probiotics

## Abstract

**Objectives:**

This double-blind, randomized, placebo-controlled clinical trial evaluated the adjuvant effects of *Bifidobacterium lactis* HN019 on the treatment of plaque-induced generalized gingivitis.

**Materials and methods:**

Sixty patients were submitted to professional supragingival scaling and prophylaxis. They were randomly assigned to test (probiotic lozenges containing *B. lactis* HN019, *n* = 30) or control (placebo lozenges, *n* = 30) groups. Lozenges were consumed twice a day for 8 weeks. Bleeding on probing (BoP), Gingival Index (GI), Plaque Index (PI), probing depth (PD), and clinical attachment level (CAL) were evaluated at baseline and after 2 and 8 weeks. Gingival crevicular fluid (GCF) was collected at baseline and at 8 weeks for analysis of the inflammatory mediators IL-1β, IL-1α, IL-8, MCP-1, and MIP-1β. Data were statistically analyzed (*p* < 0.05).

**Results:**

After 8 weeks, both groups showed reduction in the percentage of PI, with no significant difference between groups (*p* = 0.7423). The test group presented a lower percentage of BoP and a higher percentage of sites with GI ≤ 1 when compared with the control group at the end of the study (*p* < 0.0001). At 8 weeks, the test group had a greater number of patients without generalized gingivitis than the control group (20 and 11 patients, respectively; *p* < 0.05). The test group presented significantly lower levels of IL-1α, IL-1β, and MCP-1 in GCF than the control group at the end of the study (*p* < 0.05).

**Conclusion:**

The adjunct use of *B. lactis* HN019 promotes additional clinical and immunological benefits in the treatment of generalized gingivitis.

**Clinical relevance:**

*B. lactis* HN019 can be an efficient and side-effect-free adjunct strategy in the treatment of generalized gingivitis.

## Introduction

In gingivitis, there is a semi-dysbiotic state which presents resilience, and therefore, it may be difficult to return to a microbiota associated with health [1, 2]. Gingivitis can progress to periodontitis in susceptible individuals [1]. In addition to oral hygiene, genetics and nutrition are important factors which impact the host immune-inflammatory response [2–4].

There has been a great demand for alternative active agents for plaque and gingivitis control. The use of probiotics, live microorganisms that, when administered in adequate amounts, confer a health benefit on the host, has arising interest in the dental research community as an adjuvant therapy for reducing plaque and gingivitis [5]. Probiotics can modulate the local and systemic host immunoinflammatory response through the increase of anti-inflammatory cytokines and decrease of pro-inflammatory markers [6], production of beta-defensins (BD) [7], activation of toll-like receptors (TLR) [8], and infiltration of “Natural Killer” cells [9]. In addition, probiotics may produce bacteriocins and modify the bacterial environment by reducing the adhesion of pathogenic bacteria as well as preventing their establishment, multiplication, and integration without generating bacterial resistance [10, 11].

The use of different probiotic regimens in individuals with established gingivitis or experimental gingivitis models demonstrated that they can improve gingival clinical parameters [5, 12, 13], inhibit the development of gingivitis [14], promote significant reductions of periodontopathogens in subgingival biofilm and saliva [13, 15, 16], and reduce inflammatory markers of gingival crevicular fluid (GCF) [5, 17]. In fact, during the development of gingivitis, there are consistent modifications in IL-1α, IL-1β, IL-8, MCP-1, and MIP-1β levels, which may vary according to phenotype and GCF flow [18, 19]. On the other hand, some clinical trials have shown that probiotics promoted no additional benefits on plaque, parameters of gingival inflammation [15, 16, 20], and profiles of salivary microbiome [21]. In fact, recent systematic reviews and meta-analyses on the effects of probiotics on gingivitis are conflicting [22–24]. One possible reason is the significant heterogeneity among studies. It is important to emphasize that the effects of probiotic therapies on the host response are multiple and vary since they are dependent on the strain (or combination of strains) used, dosages, duration of therapy, timing of the intervention, delivery vehicle of the probiotic strain, mode of administration, and the individual pre-existing microbiome [11, 25].

In a randomized clinical trial of our research group, the effects of the probiotic strain *Bifidobacterium animalis* subsp. *lactis* HN019 (*B. lactis* HN019) as an adjunct in the treatment of generalized chronic periodontitis promoted additional clinical benefits regarding decrease in probing pocket depth, clinical attachment gain, and reduction in bleeding on probing (BoP) [26]. Additional microbiological and immunological benefits were also observed, including reduction of the pro-inflammatory cytokines IL-8 and IL-1β. Since there are no studies evaluating the effects of *B. lactis* HN019 on the management of gingivitis, we hypothesized that this probiotic therapy could be useful to reduce inflammation or restore gingival health through modulation of local inflammatory biomarkers. In this context, the purpose of this study was to evaluate the effects of the probiotic therapy with *B. lactis* HN019 as an adjunct to conventional periodontal treatment in patients with plaque-induced generalized gingivitis.

## Materials and methods

### Study population and sample size

Patients were selected from the population referred to the Periodontal Clinic at School of Dentistry of Ribeirao Preto – University of Sao Paulo (FORP/USP, Ribeirao Preto, SP, Brazil). Patients who fulfilled the inclusion criteria were invited to participate in the study. All eligible patients were thoroughly informed of the nature, potential risks, and benefits of their participation in the study and signed a Term of Informed Consent. The study protocol was reviewed and approved by the Research Ethics Committee at FORP-USP (protocol number: 68692917.5.0000.5419) and registered at Brazilian Clinical Trials Registry (protocol number: RBR-59v2yb). The study was conducted in accordance with the Helsinki Declaration of 1975, as revised in 2013.

The sample size was determined using the software Graphpad Statemate 2.0 (GraphPad Software, Inc., San Diego, CA, USA). The ideal sample size was calculated to ensure an 80% power to recognize a significant difference of 10% in BoP (δ) between the groups analyzed with a confidence interval of 95% (*α* = 0.05) and standard deviation (Σ) of 12.43% [14], considering [Zα (1.96) + Zβ (0.84)]^2^ = 7.84. The calculation was based on the following formula: *n* = {2 [(Σ)^2^/(δ)^2^]} x (Zα + Zβ)^2^. Therefore, 24 patients were required for each experimental group, totalizing 48 patients. Considering that some patients could be lost to follow-up, 60 patients were recruited.

### Inclusion and exclusion criteria

Sixty patients diagnosed with dental plaque-induced generalized gingivitis (> 30% bleeding sites) [27] were recruited. The inclusion criteria were (1) systemically healthy individuals; (2) the presence of gingival inflammation, assessed by BoP, in more than 30% of sites, with probing depths ≤ 3 mm, without radiographic bone loss and detectable attachment loss due to periodontitis; (3) the presence of a minimum of 20 fully erupted permanent teeth, excluding third molars; and (4) willingness to adhere to the study protocol. The exclusion criteria were (1) pregnant or lactating women; (2) systemic conditions that may influence the progression of periodontal diseases or the response to treatment; (3) antimicrobial, probiotic, and/or anti-inflammatory therapy in the previous 6 months; (4) history or presence of periodontitis; (5) presence of non-plaque-induced gingival disease; (6) known allergies; (7) extensive prosthetic appliances; (8) smoking; (9) legally incapacitated patients; (10) periodontal therapy in the previous 6 months; and (11) need of prophylactic antibiotic therapy for routine dental procedures.

### Allocation concealment and intervention

Patients were instructed about an effective self-performed plaque control, including information about brushing and interproximal cleaning with flossing. Before the study began, the selected individuals were identified by a numeric code. According to a random numeric table generated by a computer software, the study coordinator (F.A.C.F.) allocated each patient into one of the following groups: control (placebo; 30 patients) or test (probiotic therapy; 30 patients).

At day 0 (baseline), all patients received supragingival scaling and polishing. Supragingival scaling was performed using both hand instruments (Gracey curettes; Hu-Friedy, Chicago, IL, USA) and ultrasonic device. Supragingival prophylaxis was performed using rubber cup and prophylaxis paste. These procedures were performed by one trained periodontist (G.A.S.) who was not informed about the treatment allocation. The participants received lozenges containing probiotic (test group) or placebo (control group). In the test group, the lozenges contained 10^9^ colony-forming units (CFUs) of *Bifidobacterium animalis* subsp. *lactis* HN019 (HOWARU® Bifido LYO 40 DCU-S, Danisco USA Inc., Madison, WI, USA). Starting from the baseline, individuals were instructed to consume one lozenge twice a day (after waking up and before bedtime) for 8 weeks. During the study, they were also instructed not to consume other probiotic products, to keep the lozenges in a refrigerator, and not to use any product for chemical control of bacterial plaque.

A compounding pharmacy produced the probiotic and non-probiotic lozenges in the same format and they were packed in identical vials. The lozenges were then sent to the coordinator of the study (F.A.C.F.), who marked the code number of each participant on a set of 112 lozenges (amount to be consumed by each participant during 8 weeks), according to the experimental group assigned. The coded lozenges were given to the examiner (M.C.R.), who distributed them to the patients and did not have any access to information regarding the content of the lozenges. In addition, the patients were blinded to the content of the lozenges and the treatment assignment during the study. The study coordinator (F.A.C.F.) revealed the meaning of each code number only when the statistical analysis of the experimental data was completed.

The participants received fourteen lozenges (placebo or probiotic) per week. Once a week, they brought back the packs of lozenges that were consumed during the week and then they received new lozenges, sufficient for another week of consumption. At these visits, patients responded to a questionnaire about side effect perceptions during the consumption of lozenges. One research assistant (P.H.F.S) conducted these procedures and was also responsible for monitoring patients’ compliance in the consumption of lozenges.

Periodontal clinical parameters were evaluated at baseline and after 2 and 8 weeks using a manual periodontal probe (PCPUNC156; Hu-Friedy, Chicago, IL, USA). At baseline, as well at 8 weeks, gingival crevicular fluid (GCF) samples were collected from 8 non-contigous interproximal sites of each patient. These procedures were conducted by a single trained and calibrated examiner (Y.L.A.S.L.), who was blinded to the experimental groups of the study.

### Examiner calibration

Calibration was performed to determine the intra-examiner (Y.L.A.S.L.) reproducibility and the kappa coefficient was 93%. Ten patients (with both bleeding and non-bleeding sites upon probing) not related to this study were selected. PD, GI, and BoP were assessed using a manual periodontal probe (PCPUNC156; Hu-Friedy, Chicago, IL, USA). Each patient was evaluated on two separate occasions 48 h apart in order to obtain the intra-examiner reliability.

### Clinical monitoring

The visible Plaque Index, evaluated dichotomously (PI; [28]), and the Gingival Index (GI; [29]) were assessed at 4 sites per tooth (mesiobuccal, buccal, distobuccal, and lingual). The following clinical periodontal parameters were assessed at 6 sites per tooth (mesiobuccal, buccal, distobuccal, mesiolingual, lingual, and distolingual): (i) probing depth (PD; mm)–measured from the free gingival margin to the bottom of the gingival sulcus; (ii) clinical attachment level (CAL; mm)—measured from the cement-enamel junction to the bottom of the sulcus; and (iii) BoP, evaluated dichotomously [28]—the presence of bleeding was considered positive when occurring up to 30 s after insertion of the probe for probing depth.

### Immunological monitoring

The supragingival biofilm of the selected dental elements was removed and the sites were carefully dried with air jets and then isolated with sterile cotton rolls. GCF samples were obtained with Periopaper® strips (Oralflow Inc., Amityville, NY, USA). The strips were carefully inserted close to the margin of the gingival sulcus, remaining for a period of 30 s. The amount of total protein in each sample was determined by conventional enzyme immunoassays (ELISA) using specific kits (DCTM Protein Assay; Bio-Rad Laboratories, Inc. Berkeley, CA, USA). Cytokine levels (IL-1α, IL-1β, monocyte chemotactic protein-1 (MCP-1), macrophage inflammatory protein-1β (MIP-1β), and IL-8) were determined (pg/μl) using commercially available kits (HCYTOMAG-60 K–Milliplex^TM^ map, Merck Millipore Headquarters, Billerica, MA, USA) and the multiplexing instrument (MAGPIX^®^ analyser; Luminex Corporation, Austin, TX, USA). The concentrations of each cytokine were estimated from the standard curve using a five-parameter polynomial equation with specific software (Xponent^®^ software; Luminex Corporation).

### Statistical analysis

The mean BoP at 8 weeks was defined as the primary outcome variable. All other parameters were considered secondary outcomes. All calculations were performed by GraphPad Prism 6 (GraphPad Software, Inc., San Diego, CA, USA). Each clinical parameter was computed per participant and then averaged across patients in both groups. The significance level was set at 5% in all tests.

Normality of the distribution and the homoscedasticity of the data were analyzed by Kolgomorov Smirnoff and Bartlett test, respectively. The variables BoP, GI ≤ 1, and PI were normally distributed and the significance of differences between groups was determined using unpaired *t*-test. The variables CAL and PD presented a non-normal distribution and the significance of differences between groups was determined using Mann–Whitney test. Within-group statistically significant differences in BoP, GI ≤ 1 and PI over the course of the study were assessed by repeated measures ANOVA followed by the Bonferroni test, while CAL and PD were assessed by Friedman followed by Dunn’s post hoc test. For the comparison of the corresponding delta values (2 weeks value − initial value and 8 weeks value − initial value) between groups, unpaired *t*-test was used for BoP, GI ≤ 1, and PI, and the Mann–Whitney test was used for CAL and PD. For intragroup comparisons of the corresponding delta values (2 weeks to baseline vs. 8 weeks to baseline), paired *t*-test was used for BoP, GI ≤ 1, and PI, and the Wilcoxon test was used for CAL and PD. The significance of differences between groups for the frequency of GI scores was assessed by Kruskal–Wallis test followed by Dunn’s post hoc multiple comparisons tests. Within-group statistically significant differences in GI scores were analyzed using Friedman test followed by Dunn’s post hoc multiple comparisons test. The frequency of patients presenting gingival health/localized gingivitis or generalized gingivitis in the test and control groups at 8 weeks was assessed by the chi-square test. Gingival health was defined as < 10% bleeding sites with probing depths ≤ 3 mm in an intact periodontium or in a reduced periodontium in non-periodontitis patients [27]. The demographic characteristics of gender and the presence or absence of orthodontic appliances were compared between groups using the chi-square test. Age distribution and number of teeth were compared between groups using unpaired *t*-test. To assess the impact of the orthodontic appliance predictor on BoP, GI ≤ 1, PI, PD, and CAL outcomes at 8 weeks, a simple linear regression analysis was conducted. Total protein values were converted to pg/mL. Final cytokine levels were obtained by dividing the initial values provided by the MAGPIX® system by the total protein content in GCF samples (pg/mL). Within-group and between-group differences in the mean levels of IL-1α, IL-1β, MCP-1, MIP-1β, and IL-8 were assessed by Wilcoxon and Mann–Whitney tests, respectively.

## Results

### Clinical monitoring

**Fig. 1:**
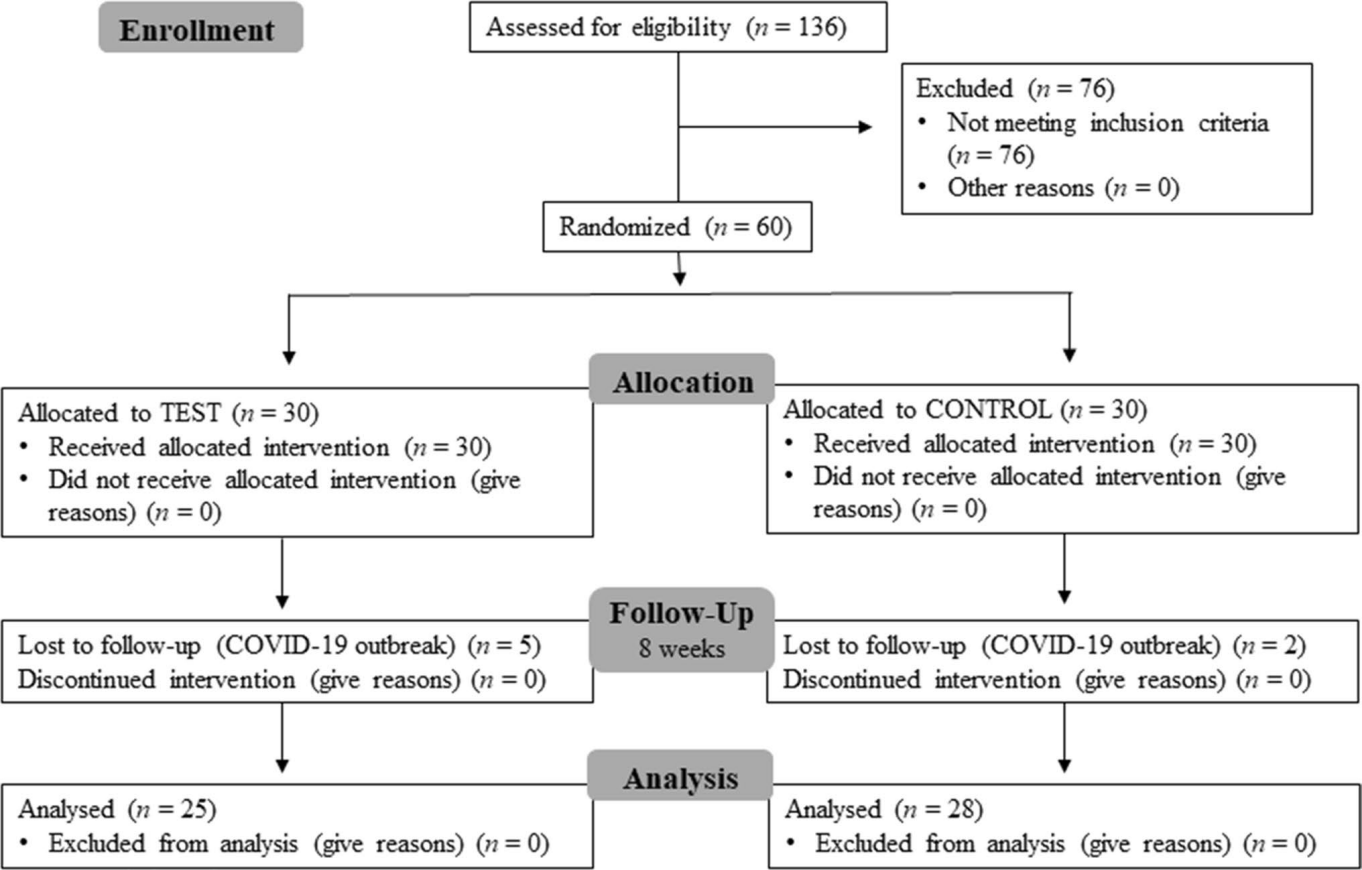
Flow chart of the study design

Figure 1 presents the flow chart of the study design. Seven ongoing patients were not followed up after baseline visit due to dental care interruption during COVID-19 pandemic. No adverse events were reported by the patients. The demographic characteristics of the sample are depicted in Table 1.

**Table 1:** Demographic characteristics of participant population at baseline

Variable	Experimental groups			*p* value*
	**Test (*n* = 25)**	**Control (*n* = 28)**	Total (*n* = 53)	
Age (mean ± SD)	23.92 ± 10.23	22.89 ± 10.90	23.38 ± 10.50	0.7260
Number of teeth (mean ± SD)	26.72 ± 2.09	26.18 ± 2.28	26.43 ± 2.18	0.3737
Gender [*n* (%)]				
Female	16 (64.00%)	18 (64.29%)	34 (64.15%)	0.9827
Male	9 (36.00%)	10 (35.71%)	19 (35.85%)	
Orthodontic appliances [*n* (%)]				
Yes	10 (40.00%)	13 (46.43%)	23 (43.40%)	0.6374
No	15 (60.00%)	15 (53.57%)	30 (56.60%)	

*SD*, standard deviation. *Comparisons between the test and control groups (chi-square test, *p* < 0.05)

**Fig. 2:**
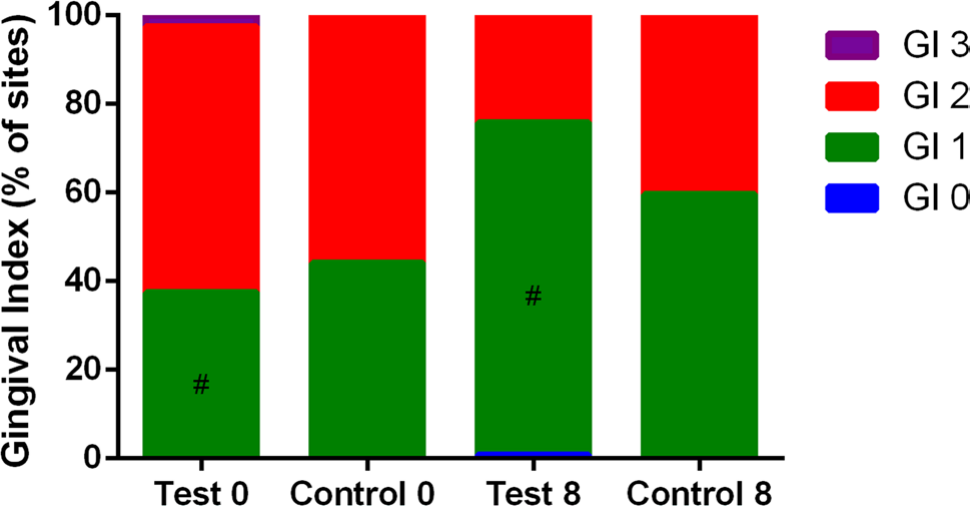
Frequency distribution of Gingival Index (GI) scores in the test and control groups at baseline and after 8 weeks. Test 0 = baseline of the test group; Control 0 = baseline of the control group; Test 8 = 8 weeks of the test group; Control 8 = 8 weeks of the control group. #Significant intragroup difference (Friedman test; Dunn’s post hoc multiple comparisons test; *p* < 0.05)

**Fig. 3:**
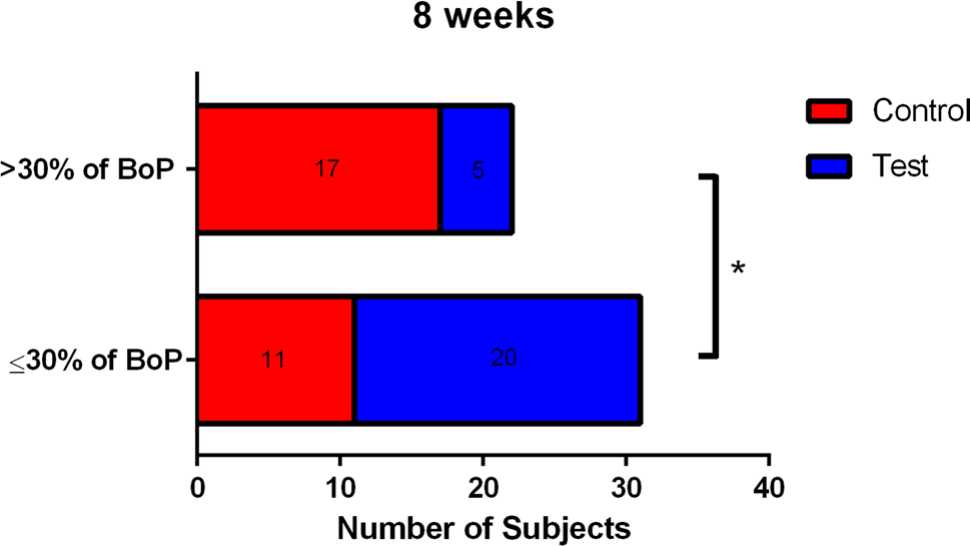
Frequency of patients of the test and control groups presenting > 30% bleeding sites (generalized gingivitis) and ≤ 30% bleeding sites (without generalized gingivitis) at 8 weeks. *Significant difference between groups (chi-square test, *p* = 0.0027)

The difference in mean BoP between groups at 8 weeks was defined as the primary outcome variable. All other parameters were considered secondary outcomes. Means and standard deviations of BoP, GI ≤ 1, PI, PD, and CAL as well as delta values are shown in Table 2. The distribution of GI scores in the test and control groups at baseline and after 8 weeks is depicted in Fig. 2. At 8 weeks, the test group presented reduced values of BoP and increased values of GI ≤ 1 when compared with the control group (*p* < 0.0001). Also, delta values (2 weeks value − initial value and 8 weeks value − initial value) show greater changes in BoP and GI ≤ 1 in the test group, when compared with the control group. At 8 weeks, it was observed that 20 patients in the test group and 11 patients in the control group reached the status of gingival health (up to 10% of sites with BoP) or localized gingivitis (10–30% of sites with BoP; Fig. 3; *p* = 0.0027).

**Table 2:** Means and standard deviations or median and interquartile range of bleeding on probing (BoP), Gingival Index (GI) ≤ 1, Plaque Index (PI), probing depth (PD), and clinical attachment level (CAL), as well as changes in these parameters at 2 and 8 weeks when compared with baseline (Δ2 − 0/Δ8 − 0) in the test and control groups at baseline and after 2 and 8 weeks

VARIABLE	TIME POINT	Experimental groups				Intergroup comparisons			
		**Test (*n* = 25)**		**Control (*n* = 28)**					
		Mean ± SD or median (IQR)	Delta ± SD or Delta (IQR)	Mean ± SD or median (IQR)	Delta ± SD or Delta (IQR)	Mean difference	CI 95%	*p* value	
								Mean or median	Delta
**BoP (%)**	Baseline	62.48 ± 19.64^a^		53.38 ± 18.02^a^		− 9.10	− 1.38 to 19.59	0.3021	
	2 weeks	30.56 ± 17.7^b^	− 31.92 ± 15.82	35.85 ± 12.57^b^	− 15.62 ± 24.45	5.29	− 3.30 to 13.89	0.2214	0.0055
	8 weeks	18.79 ± 10.39^c^	− 45.20 ± 16.53^*^	35.36 ± 12.59^b^	− 18.02 ± 16.62	16.57	− 23.21 to − 9.93	< 0.0001	< 0.0001
**GI ≤ 1 (%)**	Baseline	38.25 ± 17.07^a^		43.71 ± 18.21^a^		5.46	− 15.38 to 3.69	0.2242	
	2 weeks	63.43 ± 18.30^b^	25.18 ± 12.34	59.79 ± 13.66^b^	15.69 ± 20.20	− 3.64	− 12.67 to 5.38	0.4204	0.0426
	8 weeks	76.54 ± 12.88^c^	38.29 ± 15.61^*^	59.62 ± 10.73^b^	14.37 ± 21.75	− 16.92	10.40 to 23.43	< 0.0001	< 0.0001
**PI (%)**	Baseline	77.83 ± 19.13^a^		71.57 ± 18.34^a^		− 6.26	− 4.08 to 16.60	0.2302	
	2 weeks	55.01 ± 21.57^b^	− 22.82 ± 14.76	54.45 ± 16.64^b^	− 17.13 ± 19.86	− 0.56	− 11.32 to 10.18	0.9157	0.2393
	8 weeks	48.29 ± 20.48^b^	− 29.54 ± 16.00^*^	49.96 ± 16.40^b^	− 21.61 ± 14.37	1.67	− 11.86 to 8.50	0.7423	0.0628
**PD (mm)**	Baseline	1.98 (0.68)^d^		1.96 (0.55)^d^		− 0.02	− 0.20 to 0.24	0.8992	
	2 weeks	2.05 (0.63)^d^	− 0.15 (0.48)	1.89 (0.47)^d^	− 0.03 (0.32)	− 0.16	− 0.19 to 0.33	0.4899	0.8992
	8 weeks	1.93 (0.49)^d^	− 0.13 (0.62)	2.02 (0.40)^d^	0.04 (0.32)	0.09	− 0.31 to 0.10	0.4216	0.4899
**CAL (mm)**	Baseline	0.02 (0.28)^d^		0.02 (0.13)^d^		0.00	− 0.01 to 0.02	0.5780	
	2 weeks	0.03 (0.28)^d^	0.00 (0.02)	0.04 (0.17)^d^	0.00 (0.05)	0.01	− 0.04 to 0.03	0.7783	0.2320
	8 weeks	0.03 (0.27)^d^	0.00 (0.03)	0.03 (0.27)^d^	0.01 (0.05)	0.00	− 0.02 to 0.03	0.9321	0.2337

*SD*, standard deviation; *CI*, confidence interval; *IQR*, interquartile range. Different letters indicate significant differences in intra-group comparisons in the same clinical parameter (a, b, c—repeated measures ANOVA followed by Bonferroni post hoc test for multiple comparisons; d—Friedman’s test followed by Dunn’s post hoc test for multiple comparisons; *p* < 0.05). ^*^Significant difference in intra-group comparisons: 8-week delta values when compared to 2-week delta values in the same clinical parameter (paired *t*-test; *p* < 0.05)

Simple linear regression analysis showed that after 8 weeks of treatment, orthodontic appliances did not impact BoP (*R*^2^ = 0.347; *p* = 0.675), PI (*R*^2^ = 0.0616; *p* = 0.081) and CAL (*R*^2^ = 0.0815; *p* = 0.067), but increased PD by approximately 0.22 mm (*R*^2^ = 0.111; *p* = 0.026) and reduced the percentage of sites with GI ≤ 1 around 6.69% (*R*^2^ = 0.401; *p* = 0.040), considering the whole sample of the study.

### Immunological monitoring

**Fig. 4:**
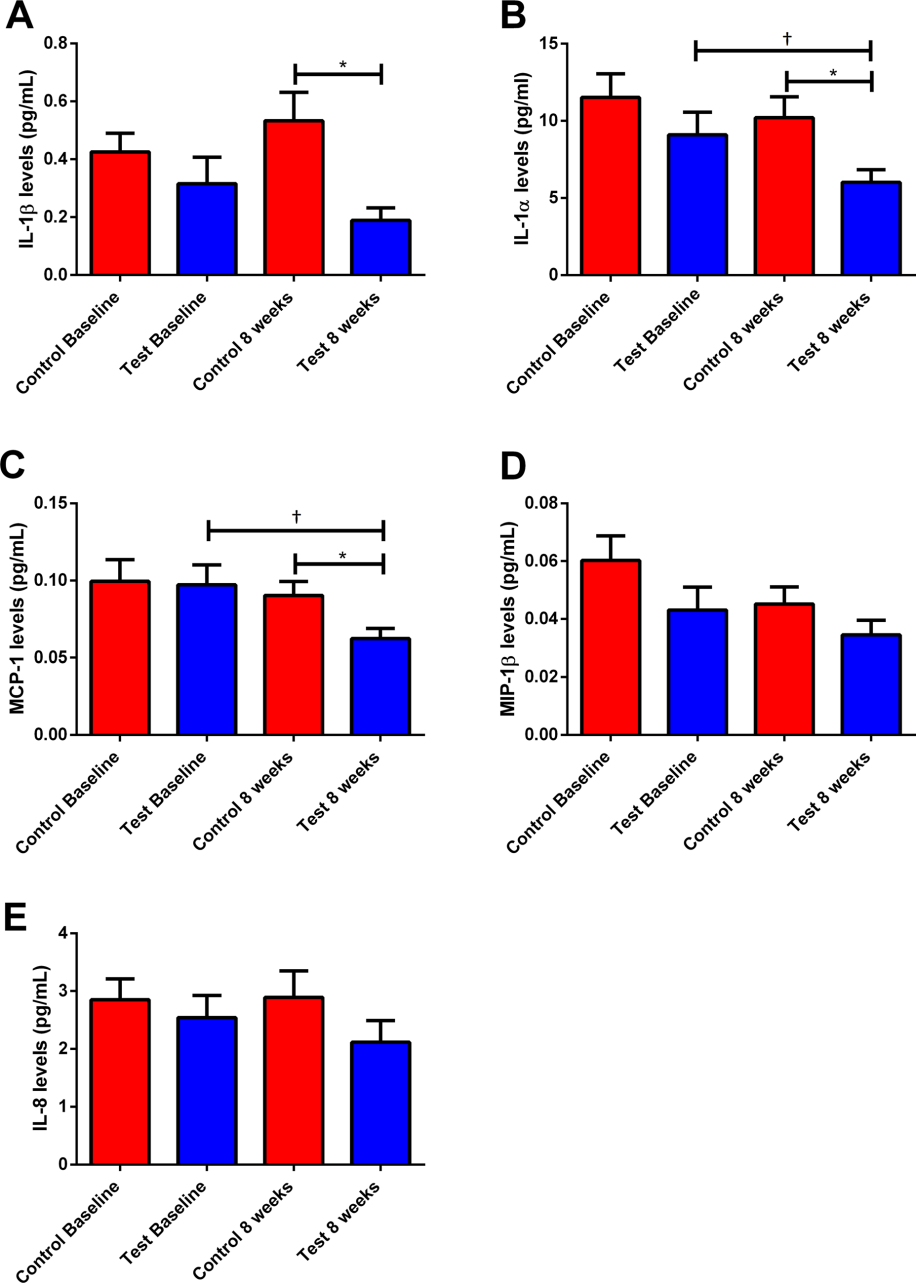
Means and standard deviations of IL-1β (**A**), IL-1α (**B**), MCP-1 (**C**), MIP-1β (**D**), and IL-8 (**E**) levels at baseline and after 8 weeks for the control and test groups. ^†^Significant intragroup difference when comparing values of baseline and 8 weeks (Wilcoxon test, *p* < 0.05). *Significant intergroup difference in the same time point (Mann–Whitney test, *p* < 0.05)

Only the test group showed a reduction in the levels of IL-1α and MCP-1 from baseline to 8 weeks (*p* < 0.05). At 8 weeks, the control group presented higher levels of IL-1β, IL-1α, and MCP-1 than the test group (*p* < 0.05; Fig. 4).

## Discussion

This double-blind, randomized, placebo-controlled clinical trial is the first one to demonstrate the potential of adjunctive administration of *B. lactis* HN019 in the treatment of generalized gingivitis. The results indicate that this probiotic therapy can provide additional benefits to conventional periodontal treatment in gingivitis patients. The probiotic group presented a significantly decreased BoP when compared with the control group at the 8-week evaluation. These findings are in disagreement with other studies that investigated the effects of probiotic therapy on gingivitis. In these trials, the administration of different probiotic regimens did not provide improvements in bleeding parameters associated with gingivitis [16, 19]. On the other hand, reductions in BoP and GI have been reported in most of the gingivitis studies using probiotics [5, 12–15, 17, 30, 31]. Besides differences in the methodologies of the studies, these contradictory results can be explained by the use of different probiotic strains, dosages, frequency, and modes of administration of probiotics [32]. Differences in the severity of gingival inflammation being treated and careful mechanical debridement before probiotic administration may also account for distinct outcomes of probiotic therapy [13, 23].

In the present study, it is worthy of note that probiotic therapy led to a major reduction in bleeding sites within 2 weeks of treatment, and the magnitude of this reduction (delta 2 weeks − baseline) was significantly greater than the one observed in the control group. This can indicate a potential for a faster resolution of gingival inflammation, which may represent an advantage for the adjunctive use of HN019. In fact, it has been hypothesized that some types of probiotic regimens may require longer consumption times so that a clinical effect can be observed [16]. On the other hand, it was demonstrated that some probiotic therapies may exert short-term positive effects but were not capable of maintaining these improvements over time [33]. In the present study, significant reductions in BoP values were maintained until the end of the trial (8-week assessments), and this may indicate a positive sustained action of *B. lactis* HN019 over time in the oral cavity.

It has been demonstrated that the effects of professional prophylaxis lasts for some weeks in gingivitis patients and then the signs of plaque and gingivitis tend to reappear [31, 34]. In the present study, the effects of professional prophylaxis leading to reduced gingival bleeding were observed in both groups at 2 weeks. However, from 2 to 8 weeks, gingival bleeding continued to drop significantly in the test group but not in the control group. It may be hypothesized that the probiotic strain used promoted immunomodulation, one of the main mechanisms of action of probiotics [35].

The immunomodulatory potential of *B. lactis* HN019 had been already demonstrated. A reduction in IL-1β levels was observed after administration of this probiotic strain in animals with ligature-induced periodontitis [36–38]. Chronic periodontitis patients consuming HN019 lozenges presented reduced levels of IL-1β and IL-8 in GCF when compared with the placebo group [26]. In the present study, a reduction in the levels of IL-1β in GCF was also observed in the test group when compared with the control group. Nevertheless, no intra- or intergroup differences were noticed in the levels of IL-8. Other randomized clinical trials evaluating probiotic therapy on gingivitis patients have not demonstrated reductions in IL-8 levels in GCF either [17, 19, 21].

It was shown that overexpression of IL-1α may be associated with cardinal features of periodontal disease, including epithelial proliferation and apical migration, loss of attachment, and destruction of cementum and alveolar bone [39]. Clinical data showed that changes in IL-1α represent a transient and reversible mediator response that co-varies with changes in clinical signs during the induction and resolution of gingivitis [18]. In the present study, only the test group presented a significant reduction in IL-1α levels from baseline to 8 weeks, reaching a significant difference when compared to the placebo group at 8 weeks. To the best of our knowledge, this is the first study to evaluate IL-1α levels in the GCF of patients with gingivitis treated with probiotics.

Decreased levels of MCP-1 were also observed in the GCF of patients who were treated with *B. lactis* HN019 in the present investigation. MCP-1 is a widely expressed chemoattractant of monocytes and macrophages [40]. In diseased gingival tissue, MCP-1 elicits the maturation of monocytes into macrophages, whose role is to destroy pathogens and secrete proinflammatory mediators, such as IL-1 and TNF-α, which is followed by a late phase of inflammation characterized by bone decomposition [36]. Thus, increased MCP-1 secretion is an indicator of periodontal damage [41]. MIP-1β is secreted by a range of inflammatory cells, including neutrophils, monocytes, and lymphocytes as well as non-inflammatory cell types at sites of inflammation, being preferentially chemotactic for the migration of CD4 + T-cell subset [42]. Nonetheless, it has been suggested that MIP-1β expressed in the gingival tissue might not increase in GCF as the intensity of inflammatory response increases, and this may explain why its levels were unaffected in the present study [42]. Another possible mechanism of action of probiotic supplementation may be to optimize and/or increase mucosal immunocompetence in healthy, immunosuppressed, or immunocompromised individuals [43]. Supporting this hypothesis, patients receiving *B. lactis* HN019 presented higher BD-3, TLR4, and cluster of differentiation-4 expressions in their oral mucosa than patients taking placebo [7].

Periodontal treatment associated with probiotic or not led to significant decreases in the mean values of PI throughout the present study. This result, which was also observed in previous gingivitis studies [44–47], can be related with an overall improvement in hygiene conditions due to the awareness of participation in a clinical study, which is known as the Hawthorne effect. Although there was no intergroup significant difference in PI, it can be observed a trend towards a greater magnitude of this reduction (delta 8 weeks − baseline) in the test group (*p* = 0.0628). Even with these improvements, in the 8-week reassessment, PI observed in both groups was not ideal, indicating a poor ability of self-performed plaque control in this population. The use of air polishing is an effective option that could have promoted different results [48]. Since fixed orthodontic treatment with multi-bracket appliances and bands is associated with increased accumulation of bacterial plaque and difficulty in its removal [49], it could be hypothesized that the presence of orthodontic appliances in some patients has impacted PI in the present study. However, they had no influence on the reported PI data at 8 weeks. Therefore, it is possible that a lack of motivation to maintain oral hygiene habits, such as not using interdental cleaning appliances, influenced the PI results in the present study.

It is important to emphasize that the impact on dental biofilm should be estimated qualitatively, irrespective of changes in the amount of dental plaque. Evidence has indicated that probiotics can exert antimicrobial activities [7, 16, 50–52] including qualitative improvements in microbiological characteristics of biofilm and/or saliva in gingivitis patients [15, 16]. It is interesting to note that the probiotic *Lactobacillus casei* Shirota led to a decrease in gingival inflammation but to an increase in PI in gingivitis patients [14]. The authors hypothesized that this result may be associated with increased availability of carbohydrates for oral microorganisms by the probiotic evaluated [14]. On the other hand, some studies demonstrated reductions in PI in gingivitis patients receiving probiotics [5, 12].

Epidemiological studies indicate that gingival inflammation is a highly prevalent condition that may affect quality of life, especially in young individuals [53]. The treatment performed in the present study promoted a shift from generalized gingivitis towards a state of localized gingivitis/gingival health in 80% of the patients in the test group versus only 39% in the control group. In this study, after 8 weeks of professional supragingival prophylaxis and supragingival scaling, the test group but not the control group presented a significant increase in the percentage of sites presenting GI ≤ 1, that is, with light signs of inflammation and without bleeding. Previous studies demonstrated that oral probiotics had significant improvement in the Gingival Index in patients with gingivitis [12, 31].

Previous meta-analyses evaluating the effects of mouthrinses with 0.12% chlorhexidine demonstrated a mean percentage reduction in gingival inflammation of 28.7% after 6 months, when considering GI [54]. Considering the same parameter in the present study, it was observed a reduction of 38.29% from baseline to 8 weeks in the test group. In fact, previous studies comparing the effects of mouthrinses containing probiotics with 0.2% chlorhexidine demonstrated that both therapies led to similar improvements in gingival inflammation and plaque reduction in gingivitis patients [55, 56]. Also, meta-analyses examining the effectiveness of dentifrices containing triclosan/copolymer after 6 to 9 months demonstrated approximately 23% reduction in gingivitis using the GI [57, 58]. The effects of HN019 on gingival inflammation observed in the present study become even more relevant when considering the drawbacks with the use of these adjunctive treatments. Triclosan is the most commonly used antiseptic in dentifrices, considering studies with periodontal diseases [59]. However, triclosan has recently been banned by the Food and Drug Administration from certain soap products due to concerns about safety and potential toxicity in humans [60]. Furthermore, chlorhexidine has been reported to have some local side effects, such as staining of the teeth and tongue, oral mucosal erosion, and taste perturbation [55, 59].

One of the limitations of this study is the short assessment period. A longer follow-up period would be required to assess the impacts of the proposed treatment over time. Microbiological analyses would also be necessary to elucidate the mechanisms of action of the probiotic therapy as an adjunct to the conventional treatment. Strong points of the present study are that many factors that can influence the effects of probiotics were well controlled, such as maintenance of lozenges’ viability and careful disruption of the biofilm prior to their administration.

## Conclusion

The oral administration of *B. lactis* HN019 promotes additional clinical and immunological benefits to mechanical debridement in the treatment of generalized gingivitis.
